# Scaling Up Species Delimitation From DNA Barcodes to Whole Organelle Genomes: Strong Evidence for Discordance Among Genes and Methods for the Red Alga *Dasyclonium*

**DOI:** 10.1111/1755-0998.14132

**Published:** 2025-06-10

**Authors:** Heroen Verbruggen, Kavitha Uthanumallian, Felix Powrie, Tara Jalali, Chiela Cremen, Maren Preuss, Sebastian Duchene, Pilar Diaz‐Tapia

**Affiliations:** ^1^ Melbourne Integrative Genomics, School of BioSciences University of Melbourne Melbourne Australia; ^2^ CIBIO, Centro de Investigação em Biodiversidade e Recursos Genéticos, InBIO Laboratório Associado Campus de Vairão, Universidade do Porto Vairão Portugal; ^3^ Department of Microbiology and Immunology, Peter Doherty Institute for Infection and Immunity University of Melbourne Melbourne Australia; ^4^ National Institute of Water and Atmospheric Research Ltd Wellington New Zealand; ^5^ Institut Pasteur Paris France; ^6^ Department of Botany Universidade de Santiago de Compostela Santiago de Compostela Spain

**Keywords:** organelle genomes, species delimitation, super‐barcodes

## Abstract

Molecular sequence data have become a ubiquitous tool for delimiting species and are particularly important in organisms where morphological traits are not informative about species boundaries. A range of statistical methods have been developed to derive species limits from molecular data, for example, by quantifying changes in branching patterns in phylogenetic trees. We aim to investigate how such methods scale up from single genes to whole organelle genomes. We gathered chloroplast genome data from 38 samples of the red algal genus *Dascyclonium* and analysed them with the popular species delimitation methods Assemble Species by Automatic Partitioning (ASAP), General Mixed Yule Coalescent (GMYC), and Poisson Tree Processes (PTP). We show extensive variation in inferred species boundaries depending on the method and dataset used. Genome‐scale analyses differed substantially between methods, with ASAP predicting the fewest species, PTP intermediate, and GMYC inferring many species. Based on a series of simulations, we identify a tendency of GMYC to overestimate species numbers as alignments increase in length, while the other two methods are not sensitive to this scaling. Gene‐by‐gene analyses show strong differences in predicted species limits, which is unexpected seeing that all genes are on a single uniparentally inherited chromosome, and highlight that choosing a particular gene as a DNA barcode has significant consequences for species diversity estimates. We show extensive cryptic diversity in the genus *Dasyclonium* and propose a consensus solution for species limits based on our combined results, enriched with biogeographic and morphological interpretations. Finally, we make recommendations for interpreting the results and improving the inferences drawn from species delimitation methods.

## 1 Introduction

Species are the currency of biodiversity, and an accurate definition of species is important for the conservation, exploitation, and technological application of biological resources. Species limits have traditionally been based on morphological comparisons among organisms, but in recent decades there has been a strong effort to design algorithms to infer species boundaries from DNA data. Among these methods are distance‐based approaches to determine barcode gaps, i.e., the difference between intra‐ and interspecific genetic divergence. These include the widely used ABGD (Automatic Barcode Gap Discovery) and ASAP (Assemble Species by Automatic Partitioning) methods (Puillandre et al. 2012, 2021). Several tree‐based methods aim to detect the transition between a speciation model in deeper parts of the tree and coalescent branching found within species, including the popular GMYC (General Mixed Yule Coalescent) and PTP (Poisson Tree Processes) methods (Fujisawa and Barraclough 2013; Zhang et al. 2013). Both the distance and tree‐based methods have largely been applied to single‐locus datasets. Another group of species delimitation methods, based on the multispecies coalescent framework, were designed for multi‐locus data and are much more computationally demanding. They include methods like BPP (Yang and Rannala 2010), Bayes Factor delimitation (Leaché et al. 2014), and SpedeSTEM (Ence and Carstens 2011).

Methods to delimit species based on molecular data are particularly important for groups where other sources of information cannot reliably infer species boundaries. These include small and morphologically simple organisms that do not offer many traits for morphological discrimination between species (the low‐morphology problem; Verbruggen et al. 2009), and groups that feature cryptic diversity due to stasis or convergence (Verbruggen 2014). Turf and epiphytic algae suffer from a combination of these issues. While these algae belong to vastly different groups (red, green and brown algae), they share their small size and a morphology consisting of creeping axes attached to the substratum by rhizoids and bearing upright axes where the reproductive structures develop (Díaz‐Tapia and Bárbara 2013). These species' small size and particular structure make them prone to harbouring cryptic diversity, with many documented examples of species that can be distinguished only with molecular tools (Díaz‐Tapia and Verbruggen 2024). For these reasons, DNA‐based species delimitation has become the norm in algae (Leliaert et al. 2014).

In this paper, we will focus on *Dasyclonium*, a red algal genus originally erected for an Australian species featuring a particular branching pattern and arrangement of sporangia (Agardh 1894). Nine more species were described or moved into this genus based on morphology, mainly from Australia and New Zealand, with three species recorded in other Pacific locations (Guiry and Guiry 2024). Molecular work on species limits in the genus has not been carried out yet, but a broader paper on the family Rhodomelaceae to which *Dasyclonium* belongs suggested that species diversity may be higher than anticipated, with four putative molecular species among specimens that corresponded to *D. incisum* in their morphology (Díaz‐Tapia et al. 2017).

In contrast to the species delimitation literature, which remains dominated by single‐marker datasets (often DNA barcodes), higher‐level phylogenetics applications have seen genome‐scale datasets derived from high‐throughput sequencing become much more common in recent years. In eukaryotes, the organelle genomes are widely used, particularly for phylogenetic studies addressing higher‐level classification or evolutionary questions, including those of red algae (Costa et al. 2016; Díaz‐Tapia et al. 2017; Muñoz‐Gómez et al. 2017; Yang et al. 2015) and many other organisms (Bernt et al. 2013; Moore et al. 2007; Wideman et al. 2020). These organelle genomes include chloroplast and mitochondrial genomes—strongly reduced remnants of the genomes of the cyanobacterial and protobacterial endosymbionts that formed these organelles.

Organelle genomes typically show uniparental (maternal) inheritance and lack recombination (Birky Jr. 1995), so in terms of genealogy, organelle genomes act as a single locus. Due to their substantial interspecific variation, particularly at silent sites, organelle genes have often been chosen for DNA barcoding and species delimitation, e.g., the mitochondrial COI (cytochrome C oxidase) in animals and the *rbc*L (RuBisCO) and *tuf*A (elongation factor Tu) genes often used in algae (Hebert et al. 2004; Saunders and McDevit 2012). Extending DNA barcoding and species delimitation to whole organelle genomes is thus a logical advancement, as the whole genome can be seen as a single locus carrying substantially more variable positions than individual DNA barcode markers. Yet their use for species delimitation has not been extensively studied. A few recent papers show promise in an animal model (Pan et al. 2019) and limitations in a few plant examples (Ji et al. 2019; Liu et al. 2021; Lv et al. 2023; Zhang et al. 2023), but no such studies are currently available for eukaryotic groups other than animals and plants. Furthermore, there is little knowledge about how the single‐locus species delimitation methods scale to genome‐scale datasets and about variability in species delimitation results between organelle genes.

The goal of this study is to carry out a detailed investigation of species limits in the red algal genus *Dasyclonium* based on chloroplast genome data. Specifically, we aim to establish how species delimitation scales up from single‐gene datasets to whole chloroplast genomes for different species delimitation methods and investigate how variability in species delimitation observed across genes relates to molecular evolutionary features of those genes.

## 2 Material and Methods

### 2.1 Samples and Sequencing

We extracted DNA from 37 samples of *Dasyclonium* that were collected from Australia and New Zealand and preserved in silica gel. The DNA extraction followed the CTAB‐based method described in Cremen et al. (2016). Libraries were prepared (Illumina VAHTS Universal DNA library preparation kit) and sequenced on the Illumina NovaSeq platform (150 PE, ca. 3 Gb). One extra previously published chloroplast genome for *Dasyclonium flaccidum* was obtained from GenBank (Díaz‐Tapia et al. 2017).

### 2.2 Assembly and Annotation

The Illumina reads were pre‐processed and assembled with Metaphor (Salazar et al. 2022), a Snakemake workflow including adapter and quality trimming with fastp and assembly with megahit (Chen et al. 2018; Li, Liu, et al. 2015; Mölder et al. 2021). Chloroplast genome contigs were identified by BLAST hits to a *Dasyclonium flaccidum* reference genome (NC_035287). For genomes that appeared complete based on the contigs nearly matching the length of the reference genome, circularity was confirmed by mapping reads across the start and end of the contigs in Geneious Prime 2023. Gene annotation was carried out by transferring annotations from the reference *Dasyclonium* genome in Geneious Prime and performing manual inspection steps to identify the most likely start and stop codons (Marcelino et al. 2016).

### 2.3 Alignments and Statistics

We included only conserved protein genes that were present in 35 or more of the 38 genomes. Following removal of stop codons and trimming of any partial sequences to conform to a complete codon set, the sequences were aligned based on their corresponding amino acid sequences with the Translation Align function in Geneious, using MAFFT 7.309 with default settings (‐‐auto) as the algorithm to align the amino acid sequences (Katoh and Standley 2013). The total alignment length, number of indels, variable, and parsimony‐informative sites were calculated for each alignment with PhyKit 1.16.0 (Steenwyk et al. 2021).

### 2.4 Species Delimitation

We applied three species delimitation algorithms (ASAP, PTP, GMYC) to all genes separately and to a genome‐scale alignment containing the concatenation of all gene alignments. For single‐gene analyses, we restricted analyses to the 98 genes that were longer than 450 nucleotides (150 amino acids), an arbitrary threshold we employed to prevent using short genes that risk having poor signals for tree inference and species delimitation. For the genome‐scale dataset, we included all 172 named genes in the concatenation, irrespective of their length. The concatenation of alignments was achieved with PhyKit and yielded an alignment of 120,492 sites. Duplicate haplotypes were removed before analysis. We opted to use ASAP instead of ABGD, as ASAP was designed to improve upon ABGD by removing the requirement for a user‐defined threshold choice.

For the ASAP species delimitation method, we used the command‐line version with default settings (Puillandre et al. 2021). For PTP, we first generated phylogenies with IQtree v.2.2.2.6 using the GTR + I + G + F model (Minh et al. 2020), then ran the Bayesian bPTP v.0.51 with default settings and rerooting on the longest branch (Zhang et al. 2013). For GMYC, we first constructed phylogenies with BEAST v.1.10.4 with the GTR + I + G model, a lognormal uncorrelated molecular clock model, a coalescent (constant size) tree prior, and default prior distributions on other parameters and hyperparameters (Suchard et al. 2018). We sampled from the posterior distribution using Markov chain Monte Carlo (MCMC) of length 1 M for individual genes and 10 M for the concatenated alignment. We summarised the posterior distribution of trees by drawing the maximum clade credibility tree from the last 50% MCMC steps and with median node heights. The single‐threshold GMYC was run on the resulting trees using the splits v.1.0.20 package in R v.4.3.1 (Fujisawa and Barraclough 2013; R Core Team 2021). Species limits were plotted onto a tree using ggtree v.3.10.1 and ggplot2 v.3.5.0 in R v.4.3.1 (Wickham 2016; Yu et al. 2017). Sequence divergences (uncorrected *p*‐values) were calculated within and between inferred species using the dist.dna function from ape v.5.7.1 in R v.4.3.1 (Paradis and Schliep 2019).

### 2.5 Scaling Up From Genes to Genomes

To investigate the scaling of species delimitation methods from genes to genomes, we simulated datasets of various sizes by subsetting the genome‐scale dataset. First, we generated 10 replicate genome‐scale alignments by concatenating the genes in a randomised order, resulting in ten unique re‐ordered versions of the data. Each of those 10 replicate alignments was then subset into 11 sub‐alignments of increasing size, ranging from 316 nt (=10^2.5^) to 100,000 nt (=10^5^) in steps of 0.25 in the exponent, allowing us to evaluate how species delimitation results change with alignment size. The resulting sub‐alignments were analysed with ASAP, PTP, and GMYC as described above, before and after removing duplicate haplotypes.

## 3 Results

### 3.1 Chloroplast Genomes

We gathered 38 chloroplast genome datasets for *Dasyclonium* species, one from GenBank and 37 newly sequenced. For 12 of the sequenced libraries, the entire chloroplast genome was contained in a single contig, and read mapping confirmed circularity for 8 of those. The remaining 4 out of these 12 libraries showed a coverage drop at the ends of the contig, but we consider these genomes complete based on their gene content, though their chromosomal conformation remains unknown. The other 26 samples returned fragmented assemblies but had high levels of gene recovery (Table S1). The 98 genes matching our criteria for inclusion are listed in Table S2.

### 3.2 Genome‐Scale Species Delimitation

**FIGURE 1:**
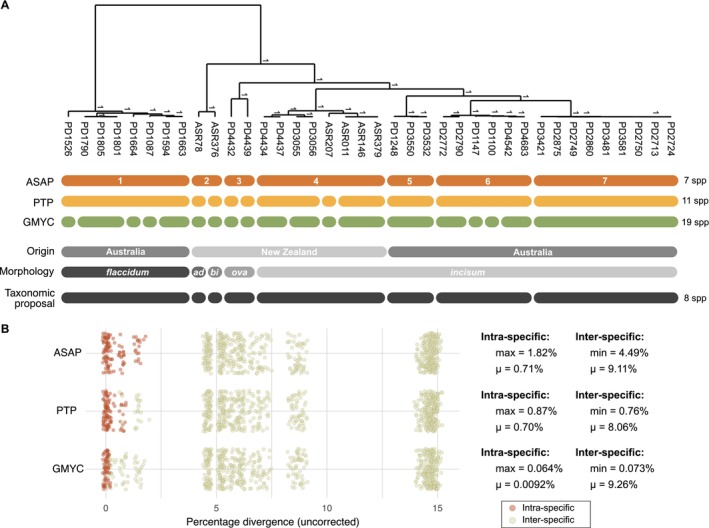
Genome‐scale species delimitation results from three methods (ASAP, PTP, GMYC). (A) Inferred species boundaries are shown against the reference tree of the samples. The samples' geographic origins and morphological identifications are indicated below the molecular species boundaries (ad = *adiantiformis*; bi = *bifurcatum*; ova = *ovalifolium*). The tree was inferred from the genome‐scale alignment with BEAST, and values at internal nodes are Bayesian posterior probabilities (shown if > 0.95). (B) Sequence divergences between samples belonging to the same (intra) or different (inter) inferred species using the three methods, along with summary statistics. Jitter was added to the points in the *x*‐ and *y*‐directions. *μ* indicates the mean.

The application of three commonly used species delimitation algorithms (ASAP, PTP, GMYC) to a genome‐scale dataset obtained by concatenating 172 gene alignments identified clear differences in inferred species boundaries, with the methods inferring between 7 and 19 species (Figure 1A). The ASAP analysis yielded the fewest, with its seven predicted species corresponding well to major lineages observed in the tree. PTP split three of these into smaller species clusters, resulting in a total of eleven predicted species. With 19 species, GMYC splits the sequences into even smaller species clusters. The distributions of sequence divergences within and between inferred species clearly reflect these different results, with ASAP having clearly demarcated intra‐ and interspecific distances (Figure 1B), and the maximum intra‐specific distance (1.82%) smaller than the minimum interspecific distance (4.49%). For PTP, some overlap was observed between the intra‐ and interspecific distances, with the maximum intra‐specific distance (0.87%) larger than the minimum interspecific distance (0.76%). This was not the case for GMYC, for which intra‐specific distances were extremely small (0.06% maximum).

The predicted species included five morphologically identified species that were distributed in either Australia or New Zealand (Figure 1). Lineage 1 corresponded to *Dasyclonium flaccidum* from Australia. Lineage 2 contained specimens morphologically identified as *D. adiantiformis* and *D. bifurcatum*, and lineage 3 had specimens of *D. ovalifolium* . Lineages 2 and 3 are from New Zealand. Finally, specimens morphologically identified as *Dasyclonium incisum* were placed in lineages 4–7, three of which are from Australia and one from New Zealand.

### 3.3 Gene‐by‐Gene Comparison

**FIGURE 2:**
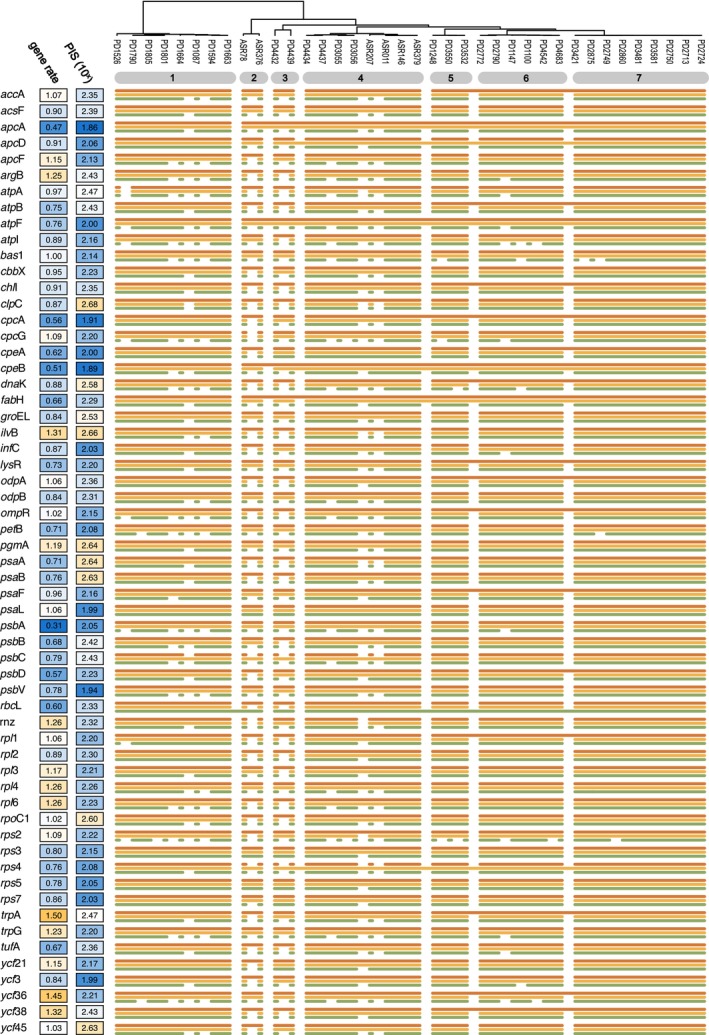
Gene‐by‐gene comparison of inferred species limits. The guide tree and the numbered grey bars at the top are taken from Figure 1 (numbers refer to the ASAP species limits) to facilitate comparison with the genome‐scale analysis. The values given on the left‐hand side are the substitution rates and the number of parsimony‐informative sites (PIS) for each gene, the latter given on a log‐10 scale. The gene rate and PIS values are also represented as colours along a blue (low) over white (intermediate) to orange (high) colour ramp. The three species delimitation methods are shown in the same colours as in Figure 1.

**FIGURE 3:**
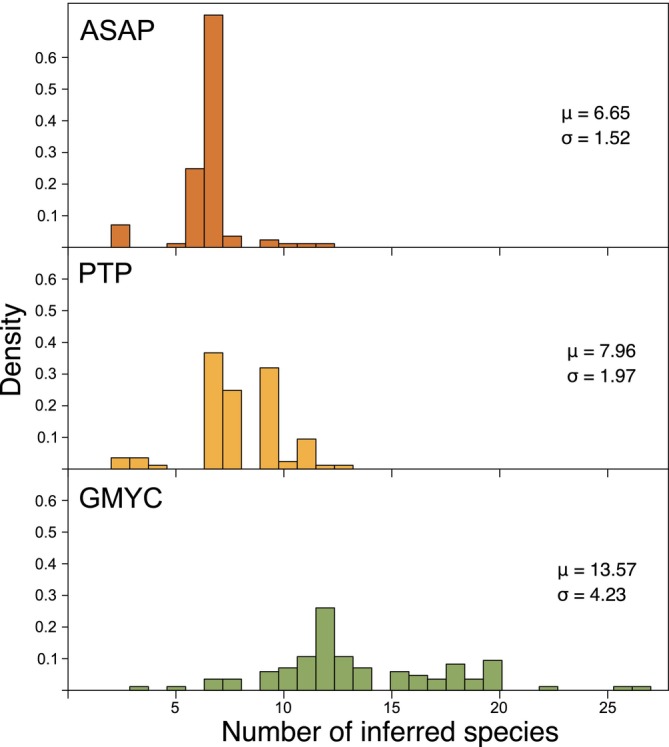
Spread of inferred species numbers from individual genes. *μ* and *σ* indicate the mean and standard deviation of the distribution of inferred species numbers.

Species delimitation carried out on 98 individual genes from the chloroplast genome showed substantial variability in the species limits across genes, with results ranging from 2 to 27 predicted species (Figure 2 and Figure S1). ASAP yielded the most consistent results, with half of all genes (49) predicting 7 species, with a small secondary peak centred on 11 species and the overall distribution having a standard deviation of 2.42 (Figure 3). PTP had more spread of values, and many genes predicted 7 (*n* = 28) or 8 (*n* = 21) species. As was the case in the genome‐scale analysis, GMYC tended to predict the largest number of species, and it had by far the widest spread of values (Figure 3).

Of the ASAP clusters that were observed in the genome‐scale analysis (also shown as the grey numbered bars in Figure 2 and Figure S1), it was mainly species 2, 3, and 4 that were subdivided (Figure 2 and Figure S1). When fewer than 7 species were predicted, it was most often species 4, 5, 6, and 7 getting merged, occasionally along with 3 and less frequently also 2.

For *rbc*L, a commonly used DNA barcode in red algae, ASAP predicted the same 7 species as in the genome‐scale analysis (Figure 2), PTP further divided lineages 2 and 3 into 2 species each, and GMYC predicted only 3 species, corresponding to ASAP lineages 1 + 2, 3, and 4–7 (Figure 2). The grouping of lineages 1 and 2 reflects the topology shown by the *rbc*L gene where these lineages are sister to one another (not shown).

To investigate the correlation of some basic molecular evolutionary features of genes with species delimitation results, we plotted the number of parsimony‐informative sites (PIS) and the rate of evolution of each gene alongside the species delimitation results (Figure 2 and Figure S1). This indicated that genes predicting very few species (e.g., PTP results for *apc*A, *apc*B, and *atp*F) were relatively conserved, with few PIS and low rates.

**FIGURE 4:**
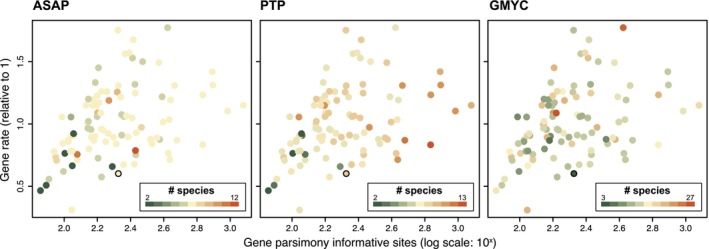
Numbers of species predicted in single‐gene analyses as a function of gene features (parsimony‐informative sites and evolutionary rate). The result for the *rbc*L gene, a traditional DNA barcode in red algae, has a black border around the point.

To visualise such relationships more comprehensively, we plotted the number of predicted species as a function of both of these gene features (Figure 4). Indeed, the lowest numbers of predicted species were systematically found in the bottom left quadrant of the plot, regardless of the method used, indicating that slowly evolving genes with few parsimony‐informative positions tend to predict fewer species. Results for higher species numbers are more scattered throughout the plots, with particularly PTP having the right‐hand side of the plot dominated by higher numbers of predicted species. Regardless of these trends, the plots also show substantial noise. Poisson regression of species numbers as a function of both predictors confirmed this, with non‐significant regression coefficients for both predictors for ASAP, a significant coefficient for PIS for PTP (*p* = 0.002), and an apparent but non‐significant coefficient for gene rate for GMYC (*p* = 0.069).

**FIGURE 5:**
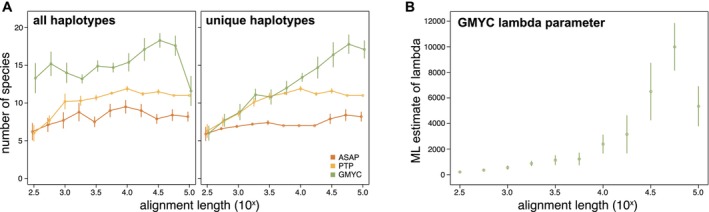
(A) Scaling of estimated species numbers with the size of the dataset, showing how methods scale from individual genes to whole organelle genomes. (B) Maximum likelihood (ML) estimates of the coalescent lambda parameter from GMYC analyses on the unique haplotype datasets. Values shown across all graphs are means of 10 replicate analyses, with the bars indicating standard error.

To investigate how species delimitation results scale up from smaller to larger datasets, we estimated species numbers on simulated alignment subsets of increasing size. This analysis shows how different methods behave as analyses are scaled up from single genes to whole genomes (Figure 5A). When all haplotypes are used in the analyses (left panel of Figure 5A), it is clear that at very small alignment sizes (up to ca. 10^2.75^ = 562 nt), inferred species numbers are smaller for ASAP and PTP, after which these methods stabilise at ca. 7–8 species for ASAP and ca. 10–11 for PTP. GMYC inferred larger numbers of species across the board.

When analyses are carried out with just one copy of each duplicated haplotype retained (right panel of Figure 5A), trends for ASAP and PTP remain similar to the analysis with all haplotypes included, though with slightly lower species numbers inferred for ASAP. GMYC, however, shows a clear increasing trend with larger numbers of species being inferred as the dataset grows. The GMYC inferred species thresholds are shown in detail in Figure S2. The maximum likelihood (ML) estimates of the coalescent branching rate (lambda.coal parameter) of the GMYC model increased markedly with alignment length (Figure 5B), as did the variation around the mean.

## 4 Discussion

Our findings reveal extensive variation in inferred species boundaries, depending on the methods and datasets employed. However, they consistently suggest that there are multiple species in *Dasyclonium*, with particularly the specimens corresponding morphologically to *D. incisum* being split into multiple species‐level lineages.

### 4.1 Method Choice Strongly Affects Genome‐Scale Species Delimitation

The discrepancies in species limits inferred from the genome‐scale data with different algorithms (Figure 1) are discouraging. If used on their own, the methods would lead to very different conclusions about *Dasyclonium* systematics, with more than double the number of species inferred with GMYC (19) than with ASAP (7). The PTP results are largely in line with ASAP, but three of the ASAP lineages (2, 3 and 4) are split into smaller species hypotheses. We note that two of these lineages (2 and 3) only contain two samples each, and while we cannot test this hypothesis based on our dataset, it would be worthwhile to investigate whether a low sample size (such as present in these lineages) would lead to more splitting in PTP.

It is interesting to consider the option that the more species‐rich GMYC result could be correct, which would indicate a substantial level of cryptic biodiversity hiding in *Dasyclonium*. However, we consider this unlikely for two reasons. First, it is poorly aligned with current knowledge about molecular species limits in red algae. The genetic distances between the GMYC species hypotheses are extremely low, with closely related species differing by only 0.073% (Figure 1). Taxonomic studies on red algae have shown divergences for the *rbc*L gene between closely related species ranging from 1% to 3% (Díaz‐Tapia et al. 2020; Freshwater et al. 2010; Savoie and Saunders 2016). Seeing that *rbc*L is among the most slowly evolving genes in the chloroplast genome (Figure 4), we consider it very unlikely that the divergences between GMYC entities, which are at least an order of magnitude smaller, can reflect realistic species boundaries. The second reason that we do not consider the genome‐scale GMYC results to be realistic is that GMYC does not appear to scale well to large datasets, as we will argue below.

### 4.2 ASAP and PTP Scale Better Than GMYC

We show here that GMYC recovers increasingly large species numbers as the alignment length is increased (Figure 5A, right panel). We attribute this to the method's dependency on inferring a species threshold from branching times in an ultrametric tree (Pons et al. 2006), which can be thought of as an inflection point in a lineages‐through‐time plot. Our data show that, as the alignment length increases, the number of unique haplotypes also increases, resulting in increasingly steep lineages‐through‐time (LTT) plots and inferred species divergence thresholds closer to zero as a consequence (Figure S2).

The underlying cause of this issue is likely that lambda, the branching rate of the coalescent process, does not get estimated well for long alignments. GMYC estimates two branching rates, one based on the coalescent for intraspecies divergence and one based on the Yule process for interspecies divergence. Both use the notation lambda, and here we refer to the intraspecies coalescent estimate. GMYC uses the Moran estimator of lambda (Nee 2001), and it is known that this parameter approaches infinity with near‐zero‐length branches in the tree, resulting in the oversplitting of species. For this reason, identical haplotypes need to be deduplicated in GMYC analyses because not doing so would result in near‐zero branch lengths (Fujisawa and Barraclough 2013).

Clearly, for the fixed set of specimens analysed here, there is only one biologically realistic coalescent branching rate. Yet we see lambda estimates ballooning for longer alignments, along with an increase in the variability of estimates across replicates (i.e., an increase of standard error in Figure 5B). In line with the reasoning above, we attribute this to an increase in near‐zero‐length branches in the trees inferred from the longer alignments resulting from the larger number of unique haplotypes in larger datasets (Figure S2). This increase in unique haplotypes is due to the chance of seeing variation between very closely related individuals increasing with the number of nucleotide positions being compared. So while these sequences are unique, they are often highly similar, differing at just a few positions across a very long alignment and yielding near‐zero‐length branches in the trees.

For analyses using all haplotypes (even identical ones), GMYC infers unrealistically large species numbers regardless of how much data are used (Figure 5A, left panel). As mentioned above, this is not recommended practice and is likely also a consequence of near‐zero‐length branches affecting the estimation of lambda.

The PTP method scales much better with increasing alignment length, with estimated species numbers converging to ca. 10–11 for alignments exceeding 10^3^ nt (1000 nt) for all haplotypes and 10^3.5^ nt (3162 nt) for unique haplotypes and remaining stable thereafter. GMYC and PTP are based on the same principle, detecting transitions between coalescent‐like branching within species and Yule‐like branching above the species level. Yet while GMYC optimises a divergence threshold on an ultrametric tree, PTP models speciation in terms of numbers of substitutions (Zhang et al. 2013). Our results clearly indicate that this approach is much less affected by the increasing number of near‐identical haplotypes as larger datasets are used. While this stability is an attractive feature, we do argue below that PTP may oversplit to some extent.

ASAP differs substantially from the two other inference methods, using hierarchical clustering of sequence distances to evaluate a barcode gap and propose species boundaries based on that. Since sequence divergences remain fairly stable when sampling increasingly large portions of the chloroplast genome, scaling up from genes to genomes did not affect the species boundaries inferred with ASAP much (Figure 5A).

All methods estimated smaller species numbers at small alignment lengths (< 10^2.75^ = 562 nt). This is an important finding, as the length of most DNA barcode amplicons is of this order of magnitude, suggesting it is plausible that current DNA barcodes systematically underestimate species numbers. This phenomenon may be further enhanced in metabarcoding, where short fragments (generally in the 200–400 bp range) are amplified directly from DNA extracted from environmental samples and sequenced on high‐throughput sequencing platforms. Our observations suggest that when applied to such short amplicons, the species delimitation methods studied here will yield a conservative estimate of species numbers.

One factor whose effect we do not evaluate here is sample size. The 38 samples presented here are a noteworthy effort at this moment in time, considering that each represents a genome‐scale dataset but remains small compared to the hundreds to thousands of samples that are often available in single‐gene species delimitation analyses. For that reason, we have focused our efforts on the comparison of the scaling of methods with data quantity across a fixed set of samples. Yet sample size is known to affect species delimitation results (Reid and Carstens 2012; Talavera et al. 2013) so, as more data become available, it will be interesting to investigate how the scaling of sampling size affects genome‐scale species delimitation.

### 4.3 Single Genes Yield a Variety of Species Hypotheses

The variability among single‐gene species limits is remarkable, ranging by an order of magnitude in the predicted species numbers. This implies that using any single chloroplast gene as a DNA barcode will, for the most part, not reflect the species limits suggested by other genes, in spite of the chloroplast genome being a single asexual locus. The features of the genes, such as the number of parsimony‐informative positions and the substitution rate, appear to correlate with the inferred species boundaries at least to some extent (Figures 2 and 4), with particular genes having lower numbers of PIS tending to have fewer species inferred from them.

We had some precautions in place (i.e., a minimum alignment length threshold of 450 nucleotides) to ensure sufficient information was present in single‐gene analyses to present a fair comparison. The minimum number of parsimony‐informative characters encountered among the genes (72) seemed very reasonable, and these genes with low PIS scores produced phylogenies in line with expectations; yet often, these would result in smaller numbers of species being recovered. It could be argued that in such situations, the methods may not be fully able to extract the biological signal due to a paucity of data.

Even though the ‘universal’ mitochondrial DNA barcode cytochrome oxidase subunit 1 (COI or *cox*1) has been used as a species delimitation in red algae (Saunders 2005), the chloroplast *rbc*L gene is another common workhorse for species‐level taxonomy in this group. The *rbc*L gene is often preferred as it amplifies more easily across divergent taxa, and it has been used as a phylogenetic marker for several decades, so many sequences are publicly available for comparative analysis. With its 7 and 9 predicted species for ASAP and PTP, respectively, studying the *rbc*L gene on its own would have led to comparable conclusions to the genome‐scale analysis, which is an encouraging result.

At 1464 nucleotide positions in *Dasyclonium*, *rbc*L is a relatively long marker, and its 212 PIS put it at the 75th percentile among all genes. All these statistics are favourable, suggesting that *rbc*L may not fall prey to the lowering of species numbers due to small alignments (cf. Figure 5A) or low information content (cf. Figure 4). We have not compared any of our results to the *cox*1 (COI) gene and consider this outside the scope of our study focused on chloroplast genomes, but the literature indicates that *cox*1 has higher levels of variability than *rbc*L (Díaz‐Tapia et al. 2020; Freshwater et al. 2010), so we would expect it to be at least as capable to detect species differences as the *rbc*L gene.

In contrast to ASAP and PTP, the GMYC analysis of *rbc*L returned a 3‐species hypothesis that is highly incompatible with the genome‐scale result and the ASAP and PTP results for *rbc*L. The alternative topology of the *rbc*L BEAST tree in which ASAP lineages 1 and 2 formed a clade is probably due to us not including an outgroup in our analyses, which results in the root position of the BEAST trees being determined by the relaxed clock model and tree prior, which is often not as consistent as using an outgroup (Huelsenbeck et al. 2002; Verbruggen and Theriot 2008). However, we do not expect this point to have a strong influence on the overall tree shape that determines the threshold of the GMYC model. We consider the *rbc*L result to be an outlier among GMYC results, which typically favoured higher species numbers than ASAP and PTP. The *rbc*L gene is among the slowest‐evolving genes in the chloroplast genome (Figures 2 and 4; Costa et al. 2016), and so the tendency of GMYC to infer smaller species numbers for slower genes may have contributed to the 3‐species hypotheses preferred by this method for *rbc*L.

### 4.4 Cryptic Diversity in *Dasyclonium*

Pinpointing the precise number of species in our *Dasyclonium* dataset based only on the analyses of molecular data is challenging due to the variability among genes and methods. To address this, we have aimed to discern the most prevailing patterns in the results that can be supported by geographic and morphological evidence. From the molecular perspective, our reasoning is based on a majority of the single‐gene ASAP and PTP analyses suggesting 7 or 8 species (Figure 3). Comparison of the single‐gene 7‐ and 8‐species results shows that they largely agree with the 7‐species genome‐scale ASAP result, albeit with occasional deviations where PTP splits either lineage 2 or 3 into two and ASAP rarely splits lineage 4 into two (Figure 2 and Figures S1, S2). Considering these outcomes alongside our interpretations of the trees, geographical and morphological considerations, we propose an 8‐species solution as a plausible taxonomic framework for *Dasyclonium* (see also Figure 1).

Of these eight species in total, four are cryptic species within the *Dasyclonium incisum* species complex (lineages 4–7). These are morphologically indistinguishable but molecularly clearly distinct. It is interesting to note that lineage 4, from New Zealand, was split into two species in some analyses. The overlapping distribution of specimens of these two sublineages suggests they may be separate sympatric species; however, the small molecular divergences and lack of morphological differentiation between them lead us to take a conservative approach and consider them a single species.

Lineage 1 corresponded to *Dasyclonium flaccidum*. This morpho‐species is morphologically very similar to *D. incisum* , but they can be distinguished based on the anatomy of the apices of determinate branches. *Dasyclonium flaccidum* has a 5‐12‐celled monsiphonous apical filament in determinate branches that is absent in *D. incisum* (Womersley 2003). Despite the differences, the taxonomic assignment of our specimens to *D. flaccidum* is not 100% certain in that monosiphonous filaments in our specimens were shorter, composed only of 2–3 cells. So, based on this, it may be possible that lineage 1 is not the actual *D. flaccidum* and that genuine *D. flaccidum* was not sampled in our study.

For lineages 2 and 3, different methods and genes suggested different solutions, with some subdividing these further. Our taxonomic proposal subdivides lineage 2 into two species, corresponding to two described species that are clearly morphologically distinct based on their branching patterns: *D. bifurcatum* and *D. adiantiformis* (Adams 1994). The two specimens of lineage 3 included in our study corresponded morphologically to *D. ovalifolium* , and they were collected from the same site, date, and habitat. Therefore, we tentatively propose to consider them as a single species. Additional studies with a larger sample size and covering the range of the species would be required to further investigate the possible existence of cryptic diversity in *D. ovalifolium* .

### 4.5 The Path Towards Super‐Barcodes

The fields of DNA barcoding and species delimitation are starting the transition from single‐gene approaches to genome‐scale datasets obtained with high‐throughput sequencing. Particularly, studies using whole organelle genomes, sometimes called super‐barcodes or ultra‐barcodes, have become more common. In animals, the limited examples available suggest that species discrimination and barcoding based on whole mitochondrial genomes have good potential. For example, a recent study of *Pachyhynobius* salamanders identified correspondence of genome‐based species with previously established species limits, alongside up to five cryptic species within *P. shangchengensis* (Pan et al. 2019). In line with ours, this study found higher numbers of species with GMYC than with other methods.

The situation in plants is quite contrasting, with several studies pointing out limited levels of taxon discrimination (Ji et al. 2019; Lv et al. 2023; Zhang et al. 2023). This seems to be due to the nature of molecular evolution in plants, with a combination of rapid radiation, incomplete lineage sorting, and hybridisation events all working against clean discrimination of species based on organelle sequences. As a consequence, the use of organelles as super‐barcodes has seen criticism in the plant research community (Hollingsworth et al. 2016). Land plants also have very low levels of among‐species variation in chloroplast genomes. For example, many barcode markers in the conifer genus *Cephalotaxus* showed < 20 PIS across the whole genus (Wang et al. 2022), and a whole plastome alignment in the flowering plant genus *Panax* showed only 2195 PIS (Ji et al. 2019). This starkly contrasts with our red algal example, where individual genes (among the subset of > 450 nucleotides long) had a minimum of 72 PIS (median 182.5, maximum 1200), and our genome‐scale dataset had 28,381 PIS.

Our work adds new information about a different group of eukaryotes, focusing on the red algae, a group where traditional DNA barcodes work well and have become the leading source of information for defining species boundaries (Díaz‐Tapia and Verbruggen 2024; Dixon and Saunders 2013). Our focus was on species delimitation rather than identification, but the clear separation of sequences into clusters (Figure 1) suggests that specimen identification based on these sequences would likely work flawlessly.

Several recent papers have used organelle genome‐level information across a set of related species to suggest specific parts of the genome that have particularly good discrimination power and develop primer sets for the amplification of such taxon‐specific DNA barcodes (Li, Yang, et al. 2015; Parks et al. 2009). While having such high‐resolution markers for particular taxa can be valuable, our work clearly shows that different organelle genes can yield contradictory species boundaries, with the most variable genes often predicting the highest numbers of species (Figure 4), in many cases leading to what we would consider to be oversplitting. This is less of a problem for species identification purposes but undesirable for species delimitation studies.

Importantly, our work also shows that the transition from single genes to whole organelle genomes for species delimitation comes with a set of challenges. Besides the practical and financial challenges involved in performing high‐throughput sequencing on many samples per species, the large incongruity of species delimitation results between methods for our genome‐scale analyses and across genes questions the value of super‐barcodes, at least using the species delimitation methods used here. Our study clearly indicates that scaling up from individual genes to whole genomes does not automatically result in straightforward interpretations. Instead, careful evaluation of the divergent results in light of methods' behaviour, and expert taxonomic judgement was needed to determine the final species hypotheses that we present.

## Author Contributions

H.V.: designed research, analysed data, wrote the paper. K.U.: performed research, analysed data. F.P.: performed research. T.J.: performed research. C.C.: performed research. M.P.: contributed resources. S.D.: contributed analytical tools. P.D.‐T.: designed research, performed research, contributed resources, wrote the paper.

## Conflicts of Interest

The authors declare no conflicts of interest.

## Supporting information


Appendix S1.

## Data Availability

Eleven annotated high‐quality genomes were submitted to GenBank (PV491985‐96) and raw reads to ENA (study PRJEB88560). Other key datasets for this study, including the contigs for all samples and alignments of all named genes, are available via Zenodo (https://doi.org/10.5281/zenodo.12691576).
