# Supplementary material for: Scaling Up Species Delimitation From DNA Barcodes to Whole Organelle Genomes: Strong Evidence for Discordance Among Genes and Methods for the Red Alga Dasyclonium
Source: Mol Ecol Resour. 2025 Jun 10;25(7):e14132. doi: 10.1111/1755-0998.14132 (PMC12415944; doi:10.1111/1755-0998.14132)

MOLECULAR ECOLOGY RESOURCES

**Supplemental Information for:**

# Scaling up species delimitation from DNA barcodes to whole organelle genomes: strong evidence for discordance among genes and methods for the red alga *Dasyclonium*

Heroen Verbruggen, Kavitha Uthanumallian, Felix Powrie, Tara Jalali,
Chiela Cremen, Maren Preuss, Sebastian Duchene, Pilar Diaz-Tapia

## Table of Contents:


| | |
|---|---|
| **Table S1:** Details of samples and their chloroplast genomes | Page 2 |
| **Table S2:** Genes included in our analyses | Page 3 |
| **Figure S1:** Comparison of inferred species limits for genes where data was unavailable for some samples | Page 4 |
| **Figure S2:** Lineages through time plots for different-sized datasets with GMYC species-level thresholds indicated. | Page 5 |
| **Figure S3:** Summary of the 7- and 8-species hypotheses inferred by ASAP and PTP. | Page 6 |

MOLECULAR ECOLOGY RESOURCES

# **Table S1.** Details of samples and their chloroplast genomes.

Collector initials are CD = Clinton Duffy, DA = David Aguirre, HV = Heroen Verbruggen, IM = Irene Middleton, JC = Joana Costa, KD = Kyatt Dixon, MB = Margaret Brookes, MP = Maren Preuss, PD = Pilar Díaz, RA = Roberta D´Archino, WN = Wendy Nelson.

| Sample ID | Collection site; date; collector | # Contigs | # Genes | Length | Coverage | GC |
|---|---|---|---|---|---|---|
| ASR011 | Kina Kina Island, Otago, South Island, New Zealand; 29/03/2017; WN & RA | 3 | 202 | 2E+05 | 563.5 | 0.266 |
| ASR78 | East of Bishop, Stewart Island, New Zealand; 01/04/2017; WN & RA | 1 | 205 | 2E+05 | 580.8 | 0.269 |
| ASR146 | Akatore, Otago, South Island, New Zealand; 29/05/2017; WN | 1 | 207 | 2E+05 | 457.6 | 0.265 |
| ASR207 | Flat Point, Wairarapa, North Island, New Zealand; 27/06/2017; WN | 1 | 206 | 2E+05 | 277.2 | 0.266 |
| ASR376 | Straggle Head, Chalky Inlet, South Island; New Zealand; 02/11/2017; WN | 1 | 208 | 2E+05 | 440.2 | 0.268 |
| ASR379 | Straggle Head, Chalky Inlet, South Island, New Zealand; 02/11/2017; WN | 1 | 206 | 2E+05 | 979.8 | 0.265 |
| PD1087 | Point Ritchie, Victoria, Australia; 27/12/2014; PD | 1 | 204 | 2E+05 | N/A | 0.281 |
| PD1100 | Killorney Beach, Victoria, Australia; 27/12/2014; PD | 9 | 197 | 2E+05 | 75.7 | 0.266 |
| PD1147 | Merri Island, Victoria, Australia; 29/12/2014; PD | 18 | 195 | 2E+05 | 122.4 | 0.268 |
| PD1248 | Rip Channel, Port Phillip Bay, Victoria, Australia; 16/01/2015; PD | 19 | 193 | 2E+05 | 78.7 | 0.266 |
| PD1526 | Prevelly, Western Australia; 12/03/2015; PD & JC | 101 | 142 | 1E+05 | 9.1 | 0.291 |
| PD1594 | Rottnest Island, Western Australia; 15/03/2015; PD & JC | 17 | 201 | 2E+05 | 244.7 | 0.283 |
| PD1663 | Point Peron, Western Australia; 18/03/2015; PD & JC | 44 | 187 | 2E+05 | 39.8 | 0.288 |
| PD1664 | Point Peron, Western Australia; 18/03/2015; PD & JC | 11 | 204 | 2E+05 | 271.9 | 0.285 |
| PD1790 | Geraldton, Western Australia; 26/03/2015; PD & JC | 15 | 205 | 2E+05 | 99.0 | 0.282 |
| PD1801 | Drummond Cove, Western Australia; 27/03/2015; PD & JC | 32 | 197 | 2E+05 | 68.7 | 0.285 |
| PD1805 | Drummond Cove, Western Australia; 27/03/2015; PD & JC | 58 | 184 | 2E+05 | 33.2 | 0.286 |
| PD2713 | Bastion Point, Victoria, Australia; 08/11/2016; PD, HV & KD | 15 | 196 | 2E+05 | 218.0 | 0.268 |
| PD2724 | Bastion Point, Victoria, Australia; 08/11/2016; PD, HV & KD | 1 | 210 | 2E+05 | 374.3 | 0.265 |
| PD2749 | Pebbly beach, Tasmania, Australia; 09/11/2017; PD & JC | 9 | 200 | 2E+05 | 200.1 | 0.267 |
| PD2750 | Pebbly beach, Tasmania, Australia; 09/11/2017; PD & JC | 2 | 208 | 2E+05 | 780.5 | 0.265 |
| PD2772 | South Gabo Harbor, Victoria, Australia; 10/11/2016; PD, HV & KD | 3 | 202 | 2E+05 | 254.6 | 0.265 |
| PD2790 | Eastern Landing, Gabo Island, Victoria, Australia; 10/11/2016; PD, HV & KD | 6 | 203 | 2E+05 | 172.0 | 0.266 |
| PD2860 | Shipwreck Creek, Victoria, Australia; 13/11/2016; PD, HV & KD | 4 | 205 | 2E+05 | 370.2 | 0.266 |
| PD2875 | Shipwreck Creek, Victoria, Australia; 13/11/2016; PD, HV & KD | 1 | 210 | 2E+05 | 691.4 | 0.266 |
| PD3055 | Princess Bay, Wellington, New Zealand; 30/06/2017; MP | 1 | 207 | 2E+05 | 183.9 | 0.265 |
| PD3056 | Princess Bay, Wellington, New Zealand; 30/06/2017; MP | 28 | 193 | 2E+05 | 268.6 | 0.273 |
| PD3421 | Binaong Bay, Tasmania, Australia; 08/11/2017; PD & JC | 15 | 201 | 2E+05 | 137.2 | 0.267 |
| PD3481 | Bicheno, Tasmania, Australia; 11/11/2017; PD & JC | 9 | 203 | 2E+05 | 168.0 | 0.266 |
| PD3532 | Bicheno, Tasmania, Australia; 11/11/2017; PD & JC | 12 | 198 | 2E+05 | 82.4 | 0.264 |
| PD3550 | Bicheno, Tasmania, Australia; 11/11/2017; PD & JC | 1 | 207 | 2E+05 | 362.0 | 0.262 |
| PD3581 | Port Arthur, Tasmania, Australia; 13/11/2017; PD & JC | 1 | 209 | 2E+05 | 369.3 | 0.265 |
| PD4432 (AKW26) | Kapowairua, North Island, New Zealand; 25/03/2018; DA | 3 | 205 | 2E+05 | 155.7 | 0.270 |
| PD4434 (AKW054) | Kapowairua, North Island, New Zealand; 27/03/2018; WN | 1 | 207 | 2E+05 | 228.0 | 0.265 |
| PD4437 (AKW022) | Kapowairua, North Island, New Zealand; 25/03/2018; IM | 3 | 205 | 2E+05 | 455.6 | 0.265 |
| PD4439 (AKW012) | Kapowairua, North Island, New Zealand; 25/03/2018; CD | 2 | 205 | 2E+05 | 261.4 | 0.270 |
| PD4542 | Gym Beach, Southern Australia; 27/10/2018; PD & MB | 5 | 204 | 2E+05 | 196.7 | 0.265 |
| PD4683 | Pondalowi Bay, Southern Australia; 30/10/2018; PD & MB | 1 | 207 | 2E+05 | 373.1 | 0.264 |

MOLECULAR ECOLOGY RESOURCES

**Table S2.** Genes included in our analyses, listing their alignment length, pairwise identity, number of parsimony-informative sites (PIs) and rate of evolution.

| Gene | Length | Identity | PIs | Rate |
|---|---|---|---|---|
| accA | 963 | 0.918 | 222 | 1.075 |
| accB | 501 | 0.8753 | 149 | 1.1956 |
| accD | 867 | 0.9295 | 184 | 0.9249 |
| acpP | 258 | 0.9193 | 59 | 0.8819 |
| acsF | 1047 | 0.9195 | 243 | 0.8968 |
| apcA | 483 | 0.9513 | 72 | 0.4655 |
| apcB | 483 | 0.939 | 89 | 0.7329 |
| apcD | 486 | 0.9145 | 115 | 0.9077 |
| apcE | 2631 | 0.8832 | 685 | 1.2339 |
| apcF | 507 | 0.9112 | 136 | 1.1496 |
| argB | 858 | 0.882 | 268 | 1.2478 |
| atpA | 1515 | 0.9294 | 292 | 0.973 |
| atpB | 1419 | 0.9293 | 270 | 0.7531 |
| atpD | 552 | 0.8982 | 153 | 0.9015 |
| atpE | 402 | 0.8991 | 112 | 1.2424 |
| atpF | 534 | 0.9353 | 101 | 0.7642 |
| atpG | 474 | 0.9215 | 110 | 1.0559 |
| atpH | 246 | 0.9535 | 38 | 0.4951 |
| atpI | 744 | 0.9275 | 146 | 0.8929 |
| bas1 | 597 | 0.917 | 139 | 0.9978 |
| carA | 1194 | 0.873 | 372 | 1.4924 |
| cbbX | 891 | 0.9156 | 171 | 0.9513 |
| cemA | 834 | 0.9142 | 212 | 1.3072 |
| chlI | 1044 | 0.9273 | 222 | 0.9106 |
| clpC | 2457 | 0.9344 | 478 | 0.8694 |
| cpcA | 486 | 0.9476 | 81 | 0.5578 |
| cpcB | 516 | 0.9416 | 90 | 0.7075 |
| cpcG | 693 | 0.919 | 159 | 1.089 |
| cpeA | 492 | 0.9324 | 99 | 0.6191 |
| cpeB | 531 | 0.9426 | 78 | 0.5084 |
| dnaK | 1875 | 0.9298 | 376 | 0.8758 |
| fabH | 1008 | 0.9173 | 195 | 0.6583 |
| ftrB | 339 | 0.9288 | 71 | 0.9699 |
| ftsH | 1866 | 0.9343 | 363 | 0.8889 |
| gltB | 4599 | 0.9116 | 1200 | 1.15 |
| groEL | 1584 | 0.922 | 338 | 0.8409 |
| ilvB | 1788 | 0.8884 | 462 | 1.3087 |
| ilvH | 534 | 0.9349 | 111 | 0.9052 |
| infB | 2274 | 0.8674 | 780 | 1.4195 |
| infC | 516 | 0.9303 | 106 | 0.8719 |
| lysR | 945 | 0.9385 | 160 | 0.7254 |
| moeB | 1083 | 0.852 | 420 | 1.7731 |
| odpA | 1035 | 0.9206 | 230 | 1.0594 |
| odpB | 972 | 0.9134 | 206 | 0.8405 |
| ompR | 732 | 0.9313 | 140 | 1.0212 |
| pbsA | 693 | 0.9117 | 151 | 0.8676 |
| petA | 960 | 0.9025 | 257 | 1.049 |
| petB | 645 | 0.9385 | 120 | 0.7126 |
| petD | 480 | 0.9266 | 109 | 0.7812 |
| petF | 300 | 0.8567 | 102 | 1.6484 |
| petG | 111 | 0.9389 | 20 | 0.7935 |
| petJ | 339 | 0.8684 | 114 | 1.4408 |
| petM | 96 | 0.9252 | 23 | 1.0214 |
| petN | 90 | 0.9617 | 10 | 0.4181 |
| petP | 192 | 0.8956 | 51 | 1.3787 |
| pgmA | 1527 | 0.883 | 437 | 1.1927 |
| preA | 972 | 0.917 | 237 | 1.0647 |
| psaA | 2256 | 0.9339 | 433 | 0.7134 |
| psaB | 2202 | 0.9242 | 424 | 0.7642 |

| Gene | Length | Identity | PIs | Rate |
|---|---|---|---|---|
| psaC | 243 | 0.956 | 38 | 0.4427 |
| psaD | 423 | 0.9102 | 96 | 1.0021 |
| psaE | 183 | 0.9165 | 44 | 0.9657 |
| psaF | 552 | 0.9059 | 145 | 0.9555 |
| psaI | 108 | 0.913 | 22 | 0.7433 |
| psaJ | 126 | 0.9526 | 20 | 0.8402 |
| psaK | 264 | 0.8913 | 78 | 1.0097 |
| psaL | 456 | 0.9265 | 97 | 1.057 |
| psb30 | 102 | 0.9387 | 13 | 0.4365 |
| psbA | 1080 | 0.9662 | 111 | 0.309 |
| psbB | 1527 | 0.9374 | 265 | 0.6771 |
| psbC | 1383 | 0.9308 | 269 | 0.7871 |
| psbD | 1053 | 0.9453 | 168 | 0.5744 |
| psbE | 252 | 0.9663 | 23 | 0.2843 |
| psbF | 132 | 0.965 | 14 | 0.3152 |
| psbH | 201 | 0.9476 | 30 | 0.6623 |
| psbJ | 117 | 0.9617 | 10 | 0.2813 |
| psbK | 135 | 0.9516 | 22 | 0.4951 |
| psbL | 114 | 0.9723 | 9 | 0.2674 |
| psbN | 129 | 0.9288 | 22 | 0.507 |
| psbT | 93 | 0.9446 | 14 | 0.5559 |
| psbV | 492 | 0.9393 | 87 | 0.779 |
| psbW | 339 | 0.9479 | 52 | 0.6118 |
| psbX | 117 | 0.9422 | 18 | 0.5444 |
| psbY | 102 | 0.9564 | 15 | 0.5815 |
| psbZ | 189 | 0.9287 | 36 | 0.7003 |
| rbcL | 1464 | 0.9522 | 212 | 0.6018 |
| rbcS | 414 | 0.936 | 79 | 0.8258 |
| rne | 1512 | 0.9156 | 331 | 0.877 |
| rnz | 675 | 0.8804 | 209 | 1.2559 |
| rpl1 | 705 | 0.9214 | 158 | 1.0597 |
| rpl11 | 426 | 0.9079 | 99 | 1.2378 |
| rpl12 | 393 | 0.8987 | 110 | 1.0454 |
| rpl13 | 447 | 0.9086 | 115 | 0.9361 |
| rpl14 | 366 | 0.9366 | 60 | 0.5633 |
| rpl16 | 393 | 0.923 | 83 | 0.8405 |
| rpl18 | 309 | 0.8959 | 93 | 1.2044 |
| rpl19 | 366 | 0.9163 | 89 | 1.0743 |
| rpl2 | 825 | 0.9157 | 198 | 0.894 |
| rpl20 | 360 | 0.9395 | 64 | 0.8254 |
| rpl21 | 312 | 0.9228 | 75 | 0.7521 |
| rpl22 | 351 | 0.9228 | 82 | 0.9772 |
| rpl23 | 303 | 0.8986 | 87 | 1.3321 |
| rpl24 | 246 | 0.8983 | 74 | 1.1294 |
| rpl27 | 258 | 0.9064 | 57 | 0.9442 |
| rpl28 | 192 | 0.925 | 39 | 0.7511 |
| rpl29 | 192 | 0.8764 | 66 | 1.2989 |
| rpl3 | 618 | 0.9018 | 162 | 1.1717 |
| rpl31 | 207 | 0.9192 | 43 | 0.8353 |
| rpl33 | 207 | 0.8881 | 55 | 1.2479 |
| rpl34 | 123 | 0.9201 | 30 | 0.8792 |
| rpl35 | 201 | 0.92 | 50 | 0.7666 |
| rpl36 | 111 | 0.9575 | 16 | 0.3938 |
| rpl4 | 657 | 0.8969 | 181 | 1.2601 |
| rpl5 | 540 | 0.918 | 114 | 0.9226 |
| rpl6 | 534 | 0.8909 | 168 | 1.2617 |
| rpl9 | 462 | 0.8709 | 154 | 1.3327 |
| rpoA | 930 | 0.9356 | 178 | 0.8584 |
| rpoB | 3360 | 0.9261 | 683 | 0.8327 |

| Gene | Length | Identity | PIs | Rate |
|---|---|---|---|---|
| rpoC1 | 1890 | 0.9138 | 395 | 1.0154 |
| rpoC2 | 3648 | 0.8933 | 964 | 1.1037 |
| rpoZ | 234 | 0.9374 | 47 | 0.9303 |
| rps1 | 783 | 0.8789 | 258 | 1.5294 |
| rps10 | 315 | 0.8926 | 97 | 1.3487 |
| rps11 | 387 | 0.937 | 74 | 0.7154 |
| rps12 | 372 | 0.9496 | 55 | 0.5189 |
| rps13 | 378 | 0.9117 | 97 | 0.854 |
| rps14 | 300 | 0.8886 | 91 | 1.1558 |
| rps16 | 240 | 0.9028 | 64 | 1.2119 |
| rps17 | 234 | 0.9217 | 55 | 0.9563 |
| rps18 | 198 | 0.9228 | 46 | 1.2255 |
| rps19 | 282 | 0.9327 | 54 | 0.7935 |
| rps2 | 702 | 0.9205 | 166 | 1.0881 |
| rps20 | 264 | 0.8949 | 70 | 0.9021 |
| rps3 | 624 | 0.9227 | 141 | 0.7995 |
| rps4 | 603 | 0.9321 | 120 | 0.7559 |
| rps5 | 510 | 0.924 | 112 | 0.7836 |
| rps6 | 315 | 0.9077 | 67 | 0.8406 |
| rps7 | 468 | 0.9249 | 106 | 0.8588 |
| rps8 | 396 | 0.931 | 87 | 0.9127 |
| rps9 | 408 | 0.9335 | 85 | 0.7258 |
| secA | 2586 | 0.8824 | 786 | 1.3127 |
| secG | 210 | 0.8873 | 63 | 1.3106 |
| secY | 1227 | 0.9139 | 272 | 0.93 |
| tatC | 726 | 0.9035 | 186 | 1.1889 |
| thiG | 828 | 0.9076 | 219 | 1.2168 |
| thiS | 210 | 0.8961 | 61 | 1.197 |
| trpA | 837 | 0.8648 | 297 | 1.5001 |
| trpG | 564 | 0.8859 | 158 | 1.2264 |
| trxA | 327 | 0.9267 | 69 | 0.7937 |
| tsf | 660 | 0.9071 | 157 | 1.1485 |
| tufA | 1227 | 0.9382 | 228 | 0.6736 |
| ycf19 | 288 | 0.9378 | 41 | 0.532 |
| ycf20 | 222 | 0.9147 | 58 | 1.0735 |
| ycf21 | 525 | 0.8902 | 149 | 1.1492 |
| ycf22 | 657 | 0.8517 | 233 | 1.5639 |
| ycf29 | 651 | 0.9095 | 168 | 0.9586 |
| ycf3 | 513 | 0.9298 | 98 | 0.843 |
| ycf33 | 195 | 0.9197 | 41 | 1.4749 |
| ycf34 | 177 | 0.8647 | 61 | 1.8763 |
| ycf35 | 411 | 0.8673 | 151 | 1.7398 |
| ycf36 | 495 | 0.8696 | 161 | 1.451 |
| ycf37 | 510 | 0.8535 | 211 | 1.7523 |
| ycf38 | 891 | 0.8644 | 267 | 1.3179 |
| ycf4 | 546 | 0.8982 | 140 | 1.1997 |
| ycf45 | 1689 | 0.8509 | 430 | 1.0346 |
| ycf46 | 1458 | 0.8949 | 389 | 1.2183 |
| ycf52 | 519 | 0.8846 | 139 | 0.9656 |
| ycf53 | 729 | 0.8778 | 261 | 1.6755 |
| ycf54 | 315 | 0.8961 | 85 | 1.0032 |
| ycf60 | 597 | 0.9426 | 112 | 0.6647 |
| ycf63 | 765 | 0.889 | 247 | 1.5717 |
| ycf65 | 297 | 0.9222 | 69 | 0.9195 |

MOLECULAR ECOLOGY RESOURCES

**Figure S1.** Comparison of inferred species limits for genes where data was unavailable for some samples.

The guide tree is as in Figure 1 and the grey numbered gray bars at the top are the ASAP species limits from the genome-scale analysis for comparison. The values given on the left hand side are the rates and the number of parsimony informative sites (PIS) for each gene, the latter given on a log-10 scale. The three species delimitation methods are shown in the same colors as in Figure 1.

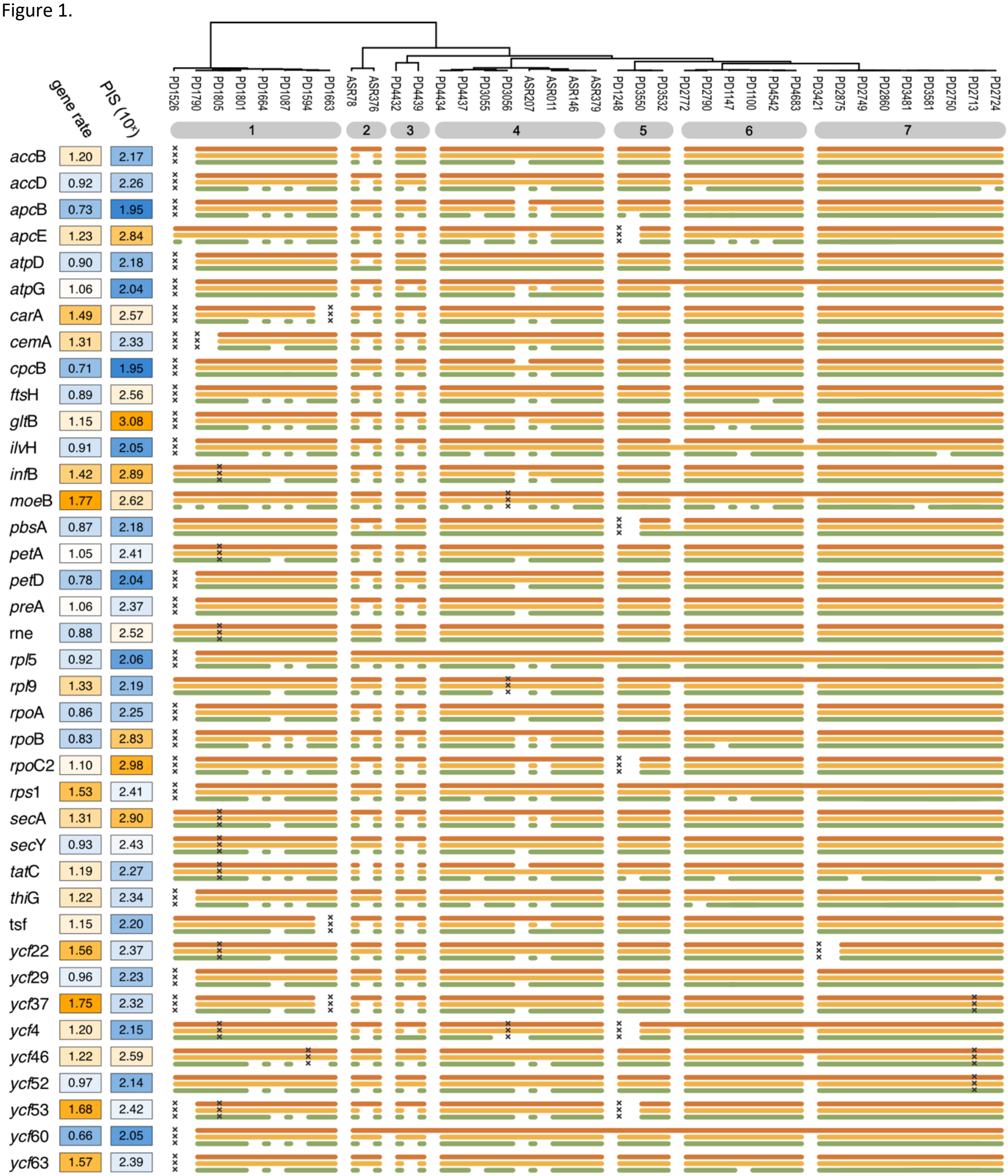

MOLECULAR ECOLOGY RESOURCES

**Figure S2.** Lineages through time plots of the BEAST phylogenies for different-sized subsets of the data, along with the species-level threshold inferred by GMYC (in red).

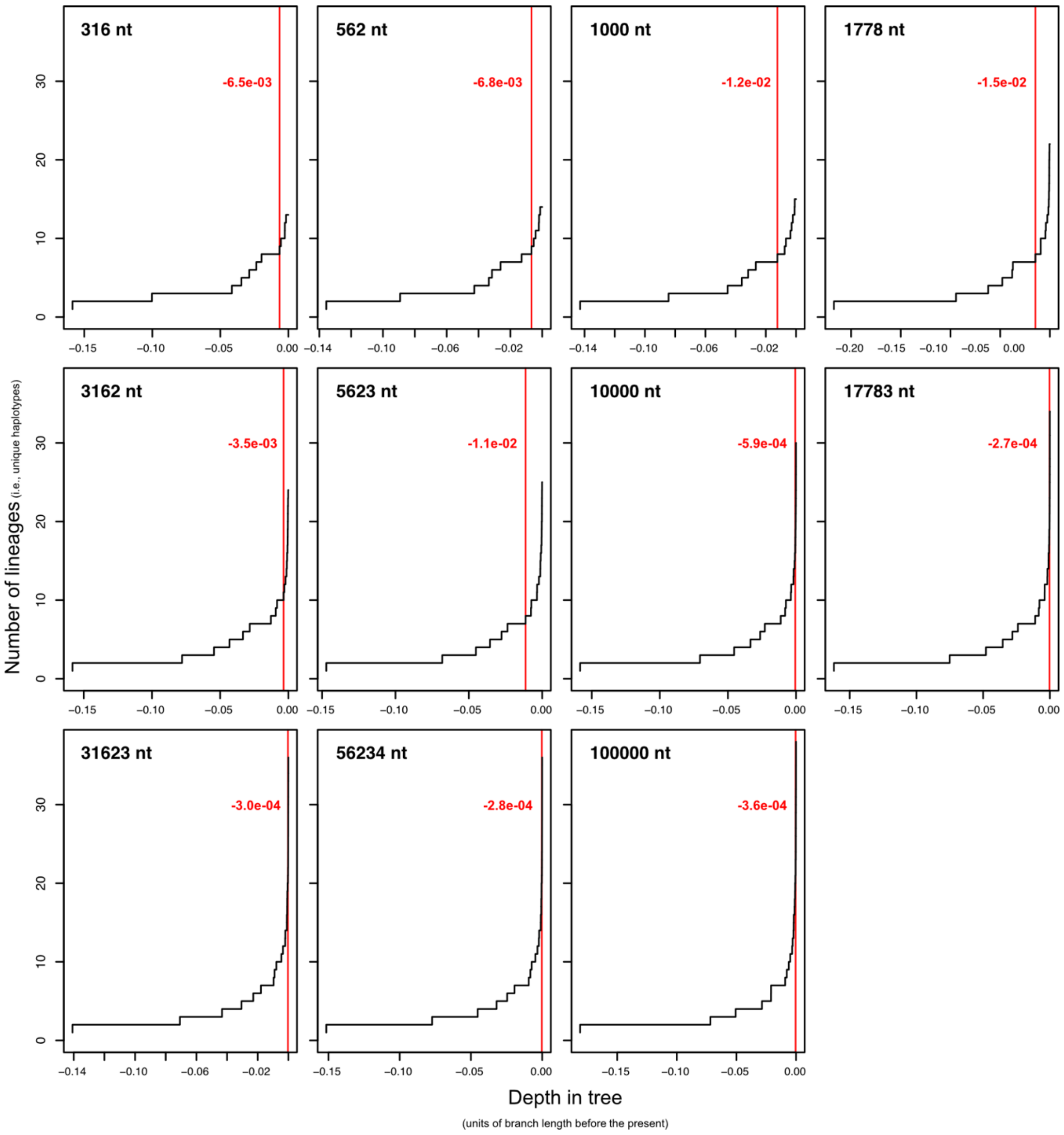

MOLECULAR ECOLOGY
RESOURCES

**Figure S3.** Summary of the 7- and 8-species hypotheses inferred by ASAP (brown) and PTP (yellow) for individual genes.

These results are extracted from Figs 2 and S1 and shown together here to visualize how these hypotheses differ from one another. GMYC results are not included here as the 7- and 8-species solution was not commonly inferred with that method.

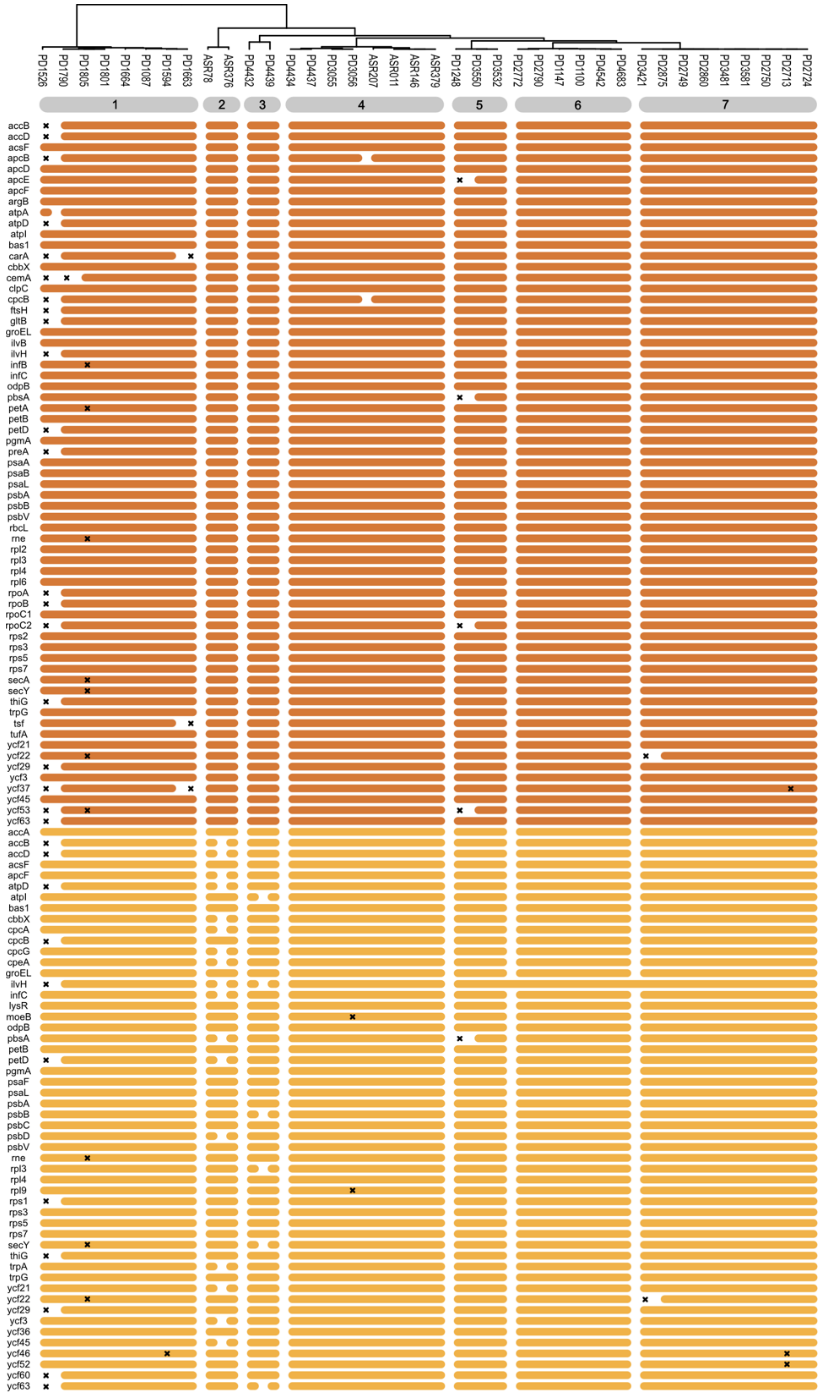

Supplement: Supplementary file 1 — Appendix S1. [file MEN-25-e14132-s001.pdf]
